# The Role of Vitamin D in Autoimmune Thyroid Diseases: From Immunomodulation to Clinical Implications

**DOI:** 10.3390/nu18020217

**Published:** 2026-01-09

**Authors:** Giulia Bendotti, Chiara Mele, Luisa Costantini, Alberto Ragni, Paola Leporati, Emilia Biamonte, Marco Gallo

**Affiliations:** 1 Endocrinology and Metabolic Diseases Unit, SS. Antonio e Biagio e Cesare Arrigo Teaching Hospital, Via Venezia 16, 15121 Alessandria, Italy; alberto.ragni@ospedale.al.it (A.R.); paola.leporati@ospedale.al.it (P.L.); emilia.biamonte@ospedale.al.it (E.B.); marco.gallo@ospedale.al.it (M.G.); 2 Endocrinology, Department of Translational Medicine, University of Piemonte Orientale, 28100 Novara, Italy; 20013221@studenti.uniupo.it (C.M.); 20058469@studenti.uniupo.it (L.C.)

**Keywords:** vitamin D, immune system, autoimmune thyroiditis, Graves’ disease

## Abstract

Vitamin D is involved in immune regulation through effects on innate and adaptive immune responses mediated by vitamin D receptor activation within immune cells. Experimental and translational studies support its role in promoting regulatory T-cell activity, modulating Th1/Th17 responses, and influencing autoantibody production. At the population level, low serum 25-hydroxyvitamin D concentrations are consistently associated with an increased risk of autoimmune diseases, including autoimmune thyroid disorders such as Hashimoto’s thyroiditis (HT) and Graves’ disease (GD), suggesting a potential preventive association. In contrast, clinical evidence from interventional studies in patients with established disease is heterogeneous. Although vitamin D supplementation has been associated with reductions in thyroid autoantibody titers in some studies—particularly in patients with HT and baseline vitamin D deficiency—consistent effects on thyroid function, disease progression, or relapse prevention have not been demonstrated. Overall, current evidence supports vitamin D deficiency as a potentially modifiable risk marker rather than a confirmed disease-modifying therapeutic target in autoimmune thyroid diseases, highlighting the need for further studies focused on clinically meaningful outcomes.

## 1. Introduction

In recent years, vitamin D has been recognized as an important regulator of immune function, with a potential role in both the development and management of autoimmune diseases. Although traditionally associated with calcium homeostasis and bone health, growing evidence shows that vitamin D directly influences innate and adaptive immune responses through activation of the vitamin D receptor (VDR) and local conversion of 25-hydroxyvitamin D into its active form, 1,25-dihydroxyvitamin D, within immune tissues [1,2,3,4]. These mechanisms modulate antigen presentation, promote regulatory T cell (Treg) development, and suppress pro-inflammatory Th1 and Th17 pathways, thereby supporting immune tolerance. Accordingly, inadequate vitamin D levels may contribute to the loss of immune regulation characteristic of autoimmunity [1,2,4]. Epidemiological data consistently associate reduced circulating 25-hydroxyvitamin D with a higher prevalence or enhanced activity of autoimmune conditions, including multiple sclerosis, type 1 diabetes, autoimmune thyroid disease, systemic lupus erythematosus, and rheumatoid arthritis [5,6,7]. Given the widespread prevalence of vitamin D deficiency, this nutrient represents a plausible environmental factor interacting with genetic predisposition and other exposures to influence autoimmune risk. Nevertheless, many observational studies are cross-sectional, limiting causal inference and raising the possibility of residual confounding and reverse causation [5,6,7,8]. Interpretation of observational data linking vitamin D status to autoimmune diseases is further complicated by the influence of systemic inflammation and chronic disease states on circulating vitamin D levels [5,6,7,8]. Reduced sunlight exposure, illness-related behavioral changes, altered vitamin D-binding protein concentrations, and inflammation-driven metabolic effects may all contribute to lower measured 25-hydroxyvitamin D concentrations in individuals with active autoimmune disease. Consequently, observational findings cannot reliably distinguish whether vitamin D deficiency contributes to autoimmune pathogenesis or reflects a consequence of disease activity.

Interventional studies evaluating vitamin D supplementation have yielded heterogeneous results. While some trials demonstrate immunomodulatory effects or reductions in surrogate markers of autoimmunity, randomized controlled trials (RCTs) often fail to show consistent benefits on clinically meaningful outcomes [5,9]. Large prevention trials, such as the VITAL study, have renewed interest in vitamin D as a potential modifier of autoimmune risk; however, the observed benefits appear to diminish after discontinuation of supplementation, underscoring uncertainty regarding durability and causality [10,11].

Although several narrative reviews and meta-analyses have examined the association between vitamin D and autoimmune thyroid diseases, challenges remain in translating this growing body of evidence into clear clinical implications. The existing literature reflects the intrinsic heterogeneity of autoimmune thyroid disorders and encompasses a wide range of study designs, populations, and outcome measures. As a result, immunological findings are often emphasized, while differences between disease entities, methodological constraints, and the clinical relevance of surrogate endpoints warrant further careful consideration.

The present review aims to provide an updated and integrative synthesis of mechanistic, epidemiological, and interventional data, with a specific focus on autoimmune thyroid diseases as distinct clinical entities. Particular attention is given to the asymmetric strength of evidence between Hashimoto’s thyroiditis (HT) and Graves’ disease (GD), the role of baseline vitamin D deficiency and disease stage, and the distinction between immunological effects (e.g., antibody modulation) and clinically meaningful outcomes. By explicitly addressing confounding, reverse causation, and heterogeneity across study designs, this review seeks to offer a pragmatic and cautious framework for interpreting the current evidence and for guiding future research rather than advocating routine therapeutic use.

## 2. Literature Search and Study Selection Approach

This narrative review is based on a comprehensive literature search conducted in PubMed/MEDLINE, Scopus, and Web of Science, including articles published up to September 2025. Search terms included combinations of “vitamin D”, “25-hydroxyvitamin D”, “autoimmune thyroid disease”, “Hashimoto’s thyroiditis”, “Graves’ disease”, “thyroid autoimmunity”, and “supplementation”. Priority was given to recent meta-analyses, randomized controlled trials, and large observational studies. Earlier mechanistic and observational studies were included when relevant to clarify biological plausibility or contextualize clinical findings. Given the heterogeneity of study designs and outcomes, evidence was synthesized qualitatively with attention to study design, baseline vitamin D status, confounder adjustment, outcome selection, and risk of bias.

## 3. Vitamin D: Biological Role and Immunomodulatory Effects

**Figure 1:**
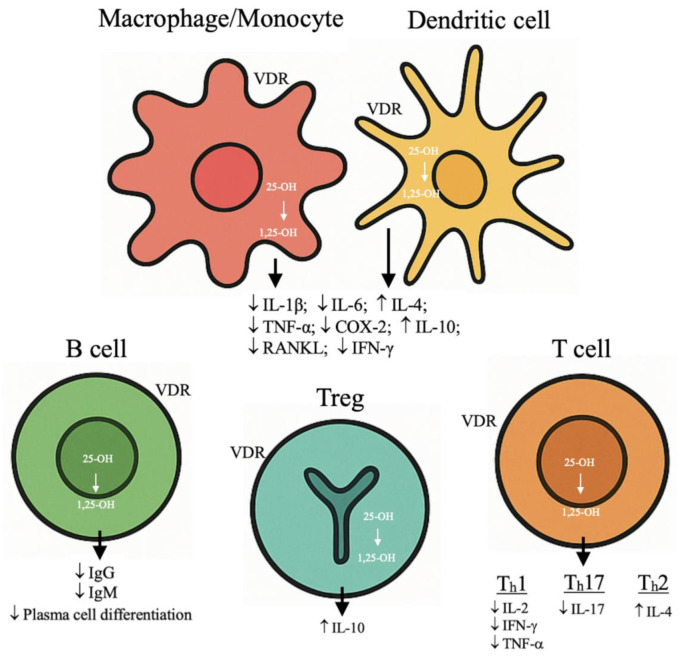
Immunomodulatory Effects of Vitamin D. This figure summarizes the proposed immunomodulatory actions of vitamin D on innate and adaptive immune cells, including effects on dendritic cells, macrophages, T lymphocyte subsets, and B cells. These mechanisms are supported by experimental and translational evidence; however, the direction and magnitude of these effects are context dependent and influenced by baseline vitamin D status, immune activation state, and experimental conditions. The pathways illustrated should not be interpreted as definitive causal relationships in clinical settings. VDR: Vitamin D Receptor. ↑ Increase; ↓ decrease.

Vitamin D_3_, the natural form of vitamin D, is synthesized in the skin from 7-dehydrocholesterol through exposure to solar ultraviolet radiation [12,13]. After its cutaneous production, vitamin D_3_ undergoes a first hydroxylation in the liver, generating 25-hydroxycholecalciferol [25(OH)D_3_]. This metabolism is then transported to the proximal renal tubules, where a second hydroxylation produces 1,25-dihydroxycholecalciferol [1,25(OH)_2_D_3_], or calcitriol, the hormonally active form of vitamin D [12,13]. One of the primary physiological roles of vitamin D is the regulation of calcium and phosphorus homeostasis, mainly by enhancing their intestinal absorption and thereby supporting adequate bone mineralization [12]. Beyond its classical skeletal actions, vitamin D is now recognized as a pleiotropic hormone involved in numerous extra-skeletal processes, including the modulation of innate and adaptive immune responses, the regulation of cell proliferation and differentiation, as well as metabolic and cardiovascular functions [14]. Importantly, these biological functions can be achieved when sufficient circulating levels of vitamin D are maintained, highlighting the need for adequate serum concentrations to ensure its physiological activity [15]. Although calcitriol is traditionally known to promote bone resorption by inducing RANKL expression in osteoblasts and driving pre-osteoclast maturation [13], the identification of VDR expression in more than 150 tissues has revealed a pleiotropic regulatory role. In fact, vitamin D influences the transcription of thousands of genes involved in physiological processes far beyond bone metabolism [13]. Immune cells—including dendritic cells, macrophages, T and B lymphocytes—express both VDR and the enzyme CYP27B1, enabling local conversion of 25(OH)D_3_ into its active form 1,25(OH)_2_D_3_ and allowing vitamin D to exert autocrine and paracrine effects within the immune microenvironment [2,16] [Figure 1]. This local synthesis enables immune cells to modulate their own responses independently of renal production [2]. Activated dendritic cells express VDR, through which vitamin D modulates their maturation and antigen-presenting capacity [17]. B lymphocytes also express VDR, particularly during differentiation into plasma cells, where vitamin D signaling contributes to the regulation of antibody production [17]. In T lymphocytes, VDR is present in both CD4^+^ and CD8^+^ subsets, with higher expression in regulatory Tregs, supporting their central role in maintaining immune tolerance [18]. Moreover, VDR and CYP27B1 expression in immune cells is upregulated by inflammatory cytokines such as IFN-γ, TNF-α, and IL-1β, linking vitamin D metabolism to immune activation [16]. Genetic polymorphisms in the VDR gene further influence immune responsiveness and susceptibility to autoimmune disorders, reinforcing the relevance of VDR in immune regulation [17]. Collectively, the widespread expression of VDR across innate and adaptive immune cells provides a strong molecular basis for the immunomodulatory actions of vitamin D [18]. Several studies indicate that vitamin D contributes to the establishment of an anti-inflammatory and tolerogenic immune microenvironment. In vitro, vitamin D suppresses macrophage- and dendritic cell-derived pro-inflammatory cytokines such as IL-1β, IL-6, IFN-γ, TNF-α, COX-2, and RANKL, while promoting the production of anti-inflammatory cytokines including IL-10 and IL-4, thereby shifting T-cell differentiation toward a Th2 phenotype [16]. Active vitamin D inhibits dendritic cell maturation and reduces the expression of MHC-II and co-stimulatory molecules, limiting antigen presentation and promoting a tolerogenic phenotype [17]. At the T-cell level, vitamin D suppresses Th1 and Th17 responses—key drivers of autoimmune inflammation—by reducing IL-2, IFN-γ, and IL-17 production, while enhancing Treg differentiation and IL-10 secretion, both essential for maintaining peripheral tolerance [16,18]. In B cells, vitamin D inhibits proliferation, plasma cell differentiation, and autoantibody production, further contributing to the prevention of autoreactivity [2]. Although supplementation studies have yielded heterogeneous results, accumulating evidence suggests that insufficient vitamin D may act as an environmental risk factor for autoimmunity and maintaining serum levels above deficiency thresholds may help modulate disease activity or progression [18,19]. Overall, vitamin D functions as a key endocrine-immune regulator capable of promoting immune tolerance and counteracting chronic autoimmune inflammation.

## 4. Vitamin D and Graves’ Disease

Graves’ disease (GD) is the most common cause of hyperthyroidism in iodine-sufficient regions, accounting for up to 80% of overt thyroid hyperfunction cases [20]. Its pathogenesis involves multiple genetic and environmental factors—including smoking, psychological stress, viral infections, and dietary iodine intake—that contribute to the loss of central and peripheral tolerance to thyroid antigens [21,22]. Hyperthyroidism in GD is driven by elevated levels of thyroid-stimulating hormone (TSH) receptor antibodies (TRAb), which bind and activate the TSH receptor on thyrocytes [22]. Both cellular and humoral immune responses participate in GD pathogenesis [23]. Beyond the well-recognized role of B lymphocytes, which produce TRAb through autoreactive clones, T-cell-mediated immunity appears to play an equally important, if not predominant, role [24,25]. Infiltration of Th1 and Treg lymphocytes has been documented in the thyroid tissue of patients with GD, accompanied by the production of Th1/Th2 cytokines and chemokines [24]. Several studies describe a predominance of Th1 activity during the active phase of the disease, with a shift toward Th2 responses in later or inactive stages [24,26]. Th1 cells are particularly active in early disease, promoting thyroid inflammation through IL-2, IFN-γ, and TNF-α secretion. These cytokine levels decrease during antithyroid therapy with methimazole and even normalize during remission or following resolution of thyrotoxicosis. Although GD was traditionally considered a Th2-predominant condition, current evidence indicates that Th1-mediated immunity predominates in early disease and during relapse [24,27]. Given the established immunomodulatory effects of vitamin D on T and B lymphocytes, its potential involvement in GD pathogenesis has been extensively investigated. However, findings remain inconsistent. Several studies report no association between circulating vitamin D levels and GD, contributing to ongoing debate [28,29,30,31]. For example, a meta-analysis by Taheriniya et al., including 42 studies and 1886 patients with autoimmune thyroid diseases [604 with GD], found significantly lower vitamin D concentrations in HT but not in GD overall, except for a reduction observed in older patients with GD, possibly influenced by age-related confounders [32]. Conversely, most studies suggest that individuals with GD are more likely to present with vitamin D insufficiency or deficiency compared with healthy controls, although prevalence varies across populations, likely due to geographic and environmental differences [33,34,35]. A meta-analysis of 27 studies conducted by Xu et al. reported that patients with GD had a significantly higher risk of vitamin D deficiency than controls (OR = 2.24, 95% CI: 1.31–3.81), supporting a potential role of vitamin D deficiency in GD susceptibility [36]. Nonetheless, vitamin D status did not correlate with clinical or biochemical indices of disease severity [37]. In contrast, Zhang et al. identified an inverse association between vitamin D levels and TRAb titers, suggesting a possible link with immunological activity, though evidence remains inconclusive [38]. Only a limited number of studies have examined vitamin D status across different stages of GD. Yasuda et al. in Japan reported significantly lower vitamin D levels in patients with active disease compared with those in remission or healthy controls [35]. A more recent study in a Thai population showed similar trends: although absolute vitamin D levels did not differ significantly, the prevalence of vitamin D deficiency was markedly higher in patients with active GD than in those in remission [21]. A negative correlation between serum vitamin D levels and free T4 was also reported in this cohort, whereas Yasuda et al. found no association between vitamin D status and TSH, fT3, or fT4 [35]. To date, no definitive evidence supports a therapeutic role for vitamin D supplementation in GD [Table 1]. A recent study reported that combined vitamin D and selenium supplementation alongside methimazole accelerated the normalization of thyroid hormone levels compared with methimazole alone [39]. However, other investigations have not replicated these findings. A recent retrospective cohort study found no significant association between vitamin D supplementation and TRAb titers or thyroid function parameters (TSH, fT4, fT3) [20]. Similar results emerged from additional studies reporting that vitamin D replacement did not significantly influence GD recurrence, relapse or remission rates [40,41].

In contrast to HT, as discussed in the following section, evidence linking vitamin D status to Graves’ disease remains inconsistent. Observational studies and meta-analyses yield heterogeneous results, and interventional trials have not demonstrated a reproducible benefit of vitamin D supplementation on remission rates, relapse prevention, or sustained control of thyroid hormone levels. These differences highlight the need for a disease-specific interpretation and caution against direct extrapolation of findings across distinct autoimmune thyroid disorders.

## 5. Vitamin D and Hashimoto’s Thyroiditis

HT is a chronic, organ-specific autoimmune disease characterized by progressive lymphocytic infiltration of the thyroid gland and the presence of circulating anti-thyroid peroxidase antibodies (TPOAb) or anti-thyroglobulin antibodies (TgAb) [48]. It represents the most common cause of hypothyroidism worldwide. The disease is primarily driven by a CD4^+^ Th1-mediated immune response that promotes gradual follicular destruction, ultimately leading to varying degrees of thyroid dysfunction [49,50]. An inverse association between low serum vitamin D concentrations and both the onset and progression of HT has been consistently documented, suggesting a pivotal role for vitamin D in modulating thyroid autoimmunity [48]. Observational studies report that more than 60% of patients with HT exhibit hypovitaminosis D, with vitamin D deficiency (<20 ng/mL) being more closely associated with HT than insufficiency (21–29 ng/mL) [30,51,52]. The inverse relationship between serum 25(OH)D and TPOAb titers was first described by Goswami and co-workers in a cohort of 642 adults and subsequently confirmed across several populations, including paediatric, premenopausal, and elderly cohorts [53]. The authors observed that lower vitamin D levels correlate with increased TSH and thyroid antibody titers, suggesting that vitamin D insufficiency may not only predispose to HT, but also accelerate its progression toward overt hypothyroidism. Mechanistically, vitamin D inhibits the production of Th1- and Th17-related proinflammatory cytokines, including IFN-γ, IL-17, and TNF-α, and promotes Tregs differentiation and IL-10 secretion [54,55,56,57,58]. Restoring the Treg/Th17 balance reduces thyroid-specific immune activation. Recent studies have shown that VDR expression is increased in thyroid-resident Tregs of patients with HT and that calcitriol enhances their suppressive capacity, particularly against TgAb production [59]. Moreover, vitamin D-mediated modulation of inflammatory cytokines may help limit tissue damage and fibrosis within the thyroid gland. Vitamin D may also regulate thyroid hormones synthesis through VDR expressed in thyrocytes, potentially modulating TSH sensitivity [48,60]. From a clinical perspective, several studies have reported an inverse association between serum vitamin D concentrations and TSH levels [34,61], supporting the hypothesis that hypovitaminosis D may contribute to progressive thyroid dysfunction. Interventional studies corroborate these immunological and biochemical observations [Table 1]. Simsek et al. demonstrated that one-month daily supplementation with 1000 IU of cholecalciferol is associated with a significant decrease in TgAb and TPOAb in vitamin D-deficient patients with HT [42]. Additional RCTs using daily doses of 2000–4000 IU or weekly doses of 50,000–60,000 IU over 8–12 weeks similarly reported reductions in thyroid autoantibodies [42,43,44,45,46,62,63]. Meta-analyses further support these findings, indicating that vitamin D supplementation can reduce TPOAb titers by 15–30%, particularly in individuals with euthyroidism or subclinical hypothyroidism [48]. Conversely, this immunological benefit appears to be attenuated in patients with overt hypothyroidism, suggesting that early correction of vitamin D deficiency may help limit antibody production before irreversible thyroid damage occurs. Nonetheless, some studies have failed to confirm a consistent association between vitamin D status and HT onset or antibody levels [29,64]. Interindividual variability may partly reflect genetic differences. Polymorphisms in the VDR gene, such as FokI, BsmI, ApaI, and TaqI are known to influence receptor function and may affect both susceptibility to HT and responsiveness to vitamin D supplementation. The FokI variant produces a shorter VDR protein with lower transcriptional activity [65], whereas BsmI, ApaI and TaqI polymorphisms modify mRNA stability or splicing efficiency, thereby altering gene expression. Several of these polymorphisms [BdmI, TaqI, FokI] have been associated with increased HT risk, with the FokI variant particularly implicated in Asian populations, supporting that the interplay between genetic and environmental determinants in HT pathogenesis [65,66]. Notably, individuals with the FokI FF genotype exhibit a greater decline in autoantibody titers after vitamin D supplementation, suggesting that genotype-specific supplementation strategies may offer clinical benefits [67].

Although polymorphisms in the vitamin D receptor gene have been associated with autoimmune thyroid disease susceptibility and differential responses to supplementation, these observations derive largely from small, population-specific studies. To date, VDR genotyping should be considered hypothesis-generating rather than clinically actionable, and genotype-guided supplementation strategies cannot be recommended outside research settings.

Taken together, current evidence indicates that vitamin D deficiency is consistently associated with the presence and immunological activity of HT, particularly in early or subclinical stages of disease. Several interventional studies report reductions in thyroid autoantibody titers following vitamin D supplementation, especially among individuals with baseline deficiency. However, these findings do not establish causality, nor do they demonstrate consistent improvements in thyroid function, prevention of disease progression, or durable long-term clinical outcomes.

Accordingly, vitamin D status should be regarded primarily as a biomarker of disease activity and a potentially modifiable correlate of thyroid autoimmunity rather than a confirmed therapeutic target. Variability in treatment response among vitamin D-deficient patients likely reflect differences in genetic susceptibility, disease stage, extent of structural thyroid damage, and environmental exposures. Future research should therefore prioritize adequately powered, genotype-stratified randomized controlled trials aimed at defining clinically relevant serum targets, clarifying underlying molecular mechanisms, and evaluating long-term effects on thyroid function and disease progression.

## 6. Clinical Implications and Therapeutic Potential

**Figure 2:**
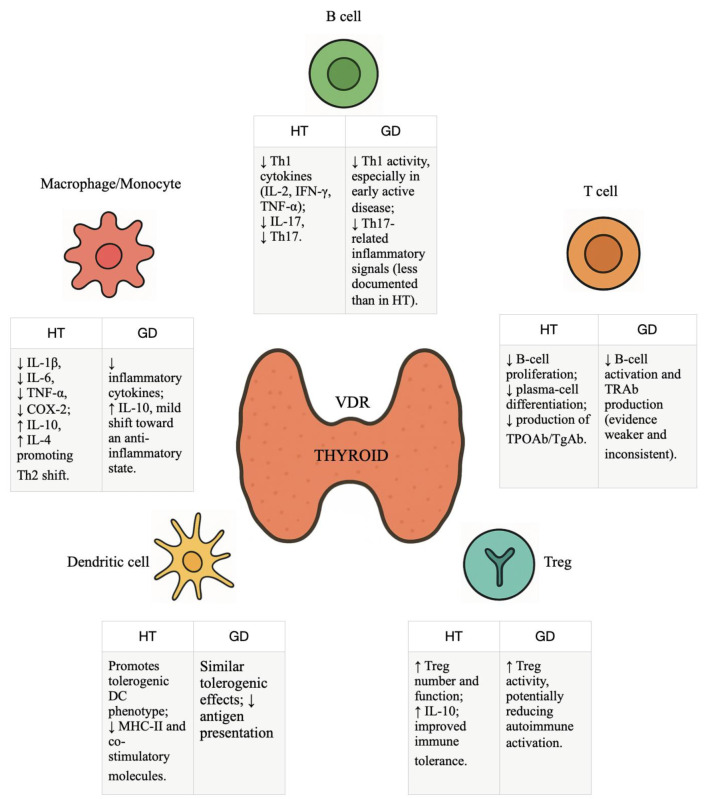
Immunological Actions of Vitamin D in Autoimmune Thyroid Diseases. This figure illustrates proposed interactions between vitamin D-mediated immune modulation and autoimmune thyroid diseases, with particular reference to Hashimoto’s thyroiditis and Graves’ disease. The depicted associations are derived from observational and interventional studies and reflect heterogeneous findings across different disease stages, populations, and study designs. Consequently, these pathways should be interpreted as conceptual frameworks rather than as evidence of uniform or causal effects. VDR: Vitamin D Receptor; HT: Hashimoto’s Thyroiditis; GD: Graves’ Disease. ↑ Increase; ↓ decrease.

Despite strong biological plausibility and consistent mechanistic evidence supporting a role for vitamin D in immune regulation, clinical studies evaluating vitamin D supplementation in autoimmune thyroid diseases (AITD) have yielded heterogeneous and sometimes conflicting results [Figure 2]. This variability reflects differences in baseline vitamin D status, supplementation dose and duration, achieved serum 25-hydroxyvitamin D concentrations, disease stage, genetic background, and outcome selection across studies [48,50].

Interventional studies remain limited in Graves’ disease. Most trials, typically enrolling fewer than 60 participants and with follow-up durations ranging from 3 to 12 months, have failed to demonstrate a reproducible benefit of vitamin D supplementation on clinically relevant outcomes such as remission rates, relapse prevention, or sustained control of thyroid hormone levels [47,68,69]. In contrast, a larger body of evidence is available for HT, where several randomized and non-randomized studies have reported reductions in thyroid autoantibody titers (TPOAb, TgAb) following vitamin D supplementation [70]. Meta-analyses indicate a mean reduction in TPOAb titers of approximately 15–30%, particularly in patients with baseline vitamin D deficiency and in euthyroid or subclinical stages of disease [48,71]. However, even in this context, effects on thyroid function parameters, including TSH, free T4, and free T3, remain inconsistent, and evidence supporting prevention of disease progression or durable clinical benefit is limited. Importantly, thyroid autoantibodies represent surrogate immunological outcomes and do not necessarily translate into clinically meaningful endpoints, such as normalization of thyroid function, reduced need for levothyroxine therapy, or improved patient-reported outcomes. Accordingly, changes in antibody titers alone should not guide clinical decision-making, especially in the absence of parallel improvements in thyroid function or disease course.

Another major source of heterogeneity relates to supplementation regimens. Across interventional trials, vitamin D has been administered using a wide range of dosing strategies, including daily doses of 1000–4000 IU, weekly regimens of 50,000–60,000 IU, or monthly bolus doses up to 200,000 IU, with treatment durations typically ranging from 8 to 24 weeks [42,44,45,46,47,69]. To date, no autoimmune thyroid disease-specific optimal serum 25-hydroxyvitamin D threshold has been established. Available evidence suggests that potential immunological effects are generally observed after correction of deficiency (commonly defined as <20 ng/mL) rather than at supraphysiological concentrations, and higher doses do not appear to confer additional benefit [12,48,69].

Notably, one randomized study reported an increase in TPOAb titers following high-dose vitamin D supplementation (50,000 IU weekly), despite modest reductions in TSH levels [72]. Although this finding should be interpreted cautiously given the small sample size and short follow-up, it raises the possibility of nonlinear or context-dependent immune effects and reinforces the need for caution against indiscriminate high-dose supplementation.

Several additional factors further complicate interpretation of interventional data. These include heterogeneity in vitamin D assays, inconsistent adjustment for confounders such as body mass index, sunlight exposure, seasonality, ethnicity, and dietary intake, as well as variability in the cut-offs used to define vitamin D deficiency and insufficiency [5,12,69]. Moreover, co-supplementation strategies—most commonly with selenium (typically 83–200 μg/day) or myo-inositol (600–2000 mg/day)—have been associated with greater reductions in thyroid autoantibody titers in some studies, particularly in HT [39,50,71]. However, the independent contribution of vitamin D in these multimodal interventions remains difficult to determine [73].

From a practical clinical perspective, vitamin D supplementation in patients with autoimmune thyroid disease should follow established indications, such as documented vitamin D deficiency, increased risk of osteoporosis or fractures, or other guideline-supported conditions, rather than being prescribed solely for immune modulation. Current evidence does not support routine screening or supplementation for autoimmune purposes alone. While correction of vitamin D deficiency may represent a reasonable adjunctive measure in selected patients—particularly those with HT—expectations regarding its impact on thyroid function, relapse prevention, or long-term disease control should remain cautious.

## 7. Conclusions

Vitamin D exerts immunomodulatory effects that are biologically plausible and consistently associated with alterations in autoimmune markers in thyroid disease, particularly in Hashimoto’s thyroiditis. However, current evidence supports vitamin D deficiency primarily as a risk marker and potential modifier of immune activity rather than a validated therapeutic target.

Supplementation may be reasonable in patients with autoimmune thyroid disease who are at risk of deficiency or have established indications, but routine use for autoimmune modulation alone is not justified. Future research should prioritize stratified, adequately powered trials focusing on baseline vitamin D deficiency, disease stage, clinically meaningful outcomes beyond autoantibodies, structural thyroid damage assessed by imaging, and the potential role of genetic background. Such an approach is essential to clarify whether targeted vitamin D interventions can meaningfully influence the clinical course of autoimmune thyroid diseases.

## Figures and Tables

**Table 1:** Interventional studies evaluating vitamin D supplementation in autoimmune thyroid diseases.

	Study	Study Design	Sample Size	Supplementation Regimen (Cholecalciferol)	Study Duration	Main Results
HT	Simsek et al., 2016 [42]	Open-label interventional study	*n* = 82	1000 IU/day	1 month	↓ TPOAb, ↓ TgAb; no consistent TSH change
HT	Chaudhary et al., 2016 [43]	Open-label RCT	*n* = 100	60,000 IU/week	8 weeks	↓ TPOAb; no significant fT4/fT3 changes
HT	Vahabi Anaraki et al., 2017 [44]	Double-blind RCT	*n* = 90	50,000 IU/week	12 weeks	↓ TPOAb, ↓ TgAb; no disease progression data
HT	Chahardoli et al., 2019 [45]	Double-blind RCT	*n* = 84	50,000 IU/week	12 weeks	↓ autoantibodies; thyroid function unchanged
HT	Villa et al., 2020 [46]	RCT	*n* = 40	2000 IU/day	6 months	↓ TSH in euthyroid HT
GD	Sheriba et al., 2017 [47]	Open-label trial	*n* = 30	2000 IU/day + antithyroid drugs	3 months	Faster biochemical improvement
GD	Cho and Chung, 2020 [40]	Retrospective cohort	*n* = 210	Vitamin D replacement (variable dose)	12 months	No effect on relapse prevention
GD	DAGMAR Trial, 2023 [41]	Double-blind RCT	*n* = 108	Vitamin D 70 μg/day	12 months	No effect on relapse or remission rates
GD	Gallo et al., 2022 [39]	RCT	*n* = 42	2000 IU/day + selenium	6 months	Faster normalization of thyroid hormones

HT: Hashimoto’s Thyroiditis; GD: Graves’ Disease; ↓ decrease.

## Data Availability

No new data were created or analyzed in this study.
